# Neonatal carnitine concentrations in relation to gestational age and weight

**DOI:** 10.1002/jmd2.12162

**Published:** 2020-09-08

**Authors:** Loek L. Crefcoeur, Monique G. M. de Sain‐van der Velden, Sacha Ferdinandusse, Mirjam Langeveld, Rose Maase, Frédéric M. Vaz, Gepke Visser, Ronald J.A. Wanders, Frits A. Wijburg, Rendelien K. Verschoof‐Puite, Peter C. J. I. Schielen

**Affiliations:** ^1^ Department of Metabolic Diseases, Wilhelmina Children's Hospital University Medical Center Utrecht Utrecht The Netherlands; ^2^ Laboratory Genetic Metabolic Diseases, Amsterdam UMC University of Amsterdam, Amsterdam Cardiovascular Sciences, Amsterdam Gastroenterology and Metabolism Amsterdam The Netherlands; ^3^ Section Metabolic Diagnostics, Department of Genetics University Medical Centre Utrecht, Utrecht University Utrecht The Netherlands; ^4^ Department of Endocrinology and Metabolism, Amsterdam UMC University of Amsterdam Amsterdam The Netherlands; ^5^ Department Biologicals, Screening and Innovation Dutch National Institute for Public Health and the Environment Bilthoven The Netherlands; ^6^ Department of Pediatrics, Emma's Children's Hospital, Amsterdam UMC University of Amsterdam Amsterdam The Netherlands; ^7^ Department for Vaccine Supply and Prevention Programmes Dutch National Institute for Public Health and the Environment Bilthoven The Netherlands; ^8^ Reference Laboratory for Neonatal Screening, Centre for Health Protection Dutch National Institute for Public Health and the Environment Bilthoven The Netherlands

**Keywords:** carnitine, LGA, newborn screening, postterm, preterm, SGA

## Abstract

**Background:**

Free carnitine has been measured in the Dutch newborn screening (NBS) program since 2007 with a referral threshold of ≤5 μmol/L, regardless of gestational age or birthweight. However, several studies suggest that carnitine concentrations may depend on gestational age and birthweight. We evaluated differences in postnatal day‐to‐day carnitine concentrations in newborns based on gestational age (GA) and/or weight for GA (WfGA).

**Methods:**

A retrospective study was performed using data from the Dutch NBS. Dried blood spot (DBS) carnitine concentrations, collected between the 3rd and 10th day of life, of nearly 2 million newborns were included. Individuals were grouped based on GA and WfGA. Median carnitine concentrations were calculated for each group. Mann‐Whitney *U* tests, and chi‐square tests were applied to test for significant differences between groups.

**Results:**

Preterm, postterm, and small for GA (SGA) newborns have higher carnitine concentrations at the third day of life compared to term newborns. The median carnitine concentration of preterm newborns declines from day 3 onwards, and approximates that of term newborns at the sixth day of life, while median concentrations of postterm and SGA newborns remain elevated at least throughout the first 10 days of life. Carnitine concentrations ≤5 μmol/L were found less frequently in SGA newborns and newborns born between 32 and 37 weeks of gestation, compared to term newborns.

**Conclusions:**

Median carnitine concentrations in NBS DBS vary with day of sampling, GA, and WfGA. It is important to take these variables into account when interpreting NBS results..

## 1 INTRODUCTION

Carnitine (3‐hydroxy‐4‐*N*‐trimethylaminobutyrate) is a quaternary amine that has an important role in energy metabolism. It facilitates the transport of activated long‐chain fatty acids into the mitochondrion where they can be degraded via beta‐oxidation.^1^ Long‐chain fatty acids either derived from exogenous, dietary sources, or generated endogenously are readily activated to their coenzyme A (CoA) esters, which explain why the bulk of fatty acids is present in cells as acyl‐CoAs. Importantly, acyl‐CoAs are not able to enter the mitochondrion and instead need to be converted into the corresponding acylcarnitine as mediated by the enzyme carnitine palmitoyltransferase 1. Acylcarnitines can enter the mitochondrion in exchange for free carnitine via the mitochondrial carnitine acylcarnitine carrier after which the acylcarnitine can be reconverted back into the original acyl‐CoA and subsequently beta‐oxidized. Apart from the important role of carnitine in mitochondrial beta‐oxidation, carnitine also plays a major role in peroxisomal beta‐oxidation and CoA homeostasis.

Several inborn errors of metabolism result in abnormal plasma acylcarnitine profiles, which can be used as a sensitive diagnostic first‐tier test.^1^ In 2007, the Dutch newborn screening (NBS) program was expanded with several of these inborn errors of metabolism. In order to perform a reliable screen, the free carnitine (ie, nonacylated carnitine) concentration should be sufficiently high, as low carnitine concentrations hinder acylcarnitine formation, and screening profiles thus cannot be reliably interpreted. For this reason, free carnitine is also measured and evaluated. A free carnitine concentration >5 μmol/L in the dried blood spot (DBS) is used as cutoff for reliable interpretation of the acylcarnitine profile. In newborns with a lower value, the measurement is repeated in a new DBS and if low carnitine levels persist they are referred to a metabolic center. In this center, potential causes for low carnitine concentrations in the newborn, such as primary carnitine deficiency (PCD) or secondary deficiency, either due to another metabolic disorder, or subsequent to maternal carnitine deficiency will be investigated. The referral threshold of ≤5 μmol/L is used for all newborns, regardless of gestational age (GA), birthweight, or day of sampling.

Several studies, however, have shown that normal free carnitine concentrations depend on GA, weight for GA (WfGA), and day of sampling. In 1980, higher free carnitine concentrations at birth were reported in preterm infants of 30 to 36 weeks of gestation.^2^ This finding was reproduced in several other studies.^3^, ^4^, ^5^, ^6^, ^7^ Higher free carnitine concentrations in infants that are small for GA (SGA) have also been reported.^7^, ^8^ Only one study assessed the course of the carnitine concentration during the first weeks of life in preterm infants, comparing newborns with a GA of 22 to 27 and 28 to 31 weeks.^9^ In both groups, a rapid decrease in carnitine concentration within the first 7 days of life was observed. A detailed overview of the data from previous studies can be found the Supporting Information (Table S1). However, data on the day‐to‐day free carnitine concentrations in preterm and/or SGA infants compared to term infants during the first week of life are still lacking.

The Dutch screening protocol allows for a rather wide sampling window, namely, 72 to 168 hours after birth. Between 2007 and 2017, 96.9% of NBS DBS samples were collected within that window. Thus, the time‐ and GA‐dependency of free carnitine levels is relevant for correct interpretation of screening results. We therefore examined the day‐to‐day changes in carnitine concentrations in NBS bloodspots of infants of various GAs and birthweights during the 3rd up to and including 10th day of life.

## 2 METHODS

### 2.1 Study population

Data were extracted retrospectively from the registry used by the Dutch National Institute for Public Health and the Environment, Department for Vaccine Supply and Prevention Programmes (RIVM‐DVP). The following data were collected anonymously for each carnitine measurement that was performed between January 2007 and December 2017: sex, GA at birth, birthweight, carnitine concentration, age when DBS sample was taken (sample day) and registered remarks concerning reliability of the DBS (eg, insufficient filling of blood spot). Ethics approval was not required, as no identifying information was acquired. Data retrieval was approved for by the Commission Data applications Praeventis of the RIVM‐DVP.

### 2.2 Inclusion and exclusion of samples

Samples obtained between 72 and 264 hours after birth were included, in order to represent the most common period for NBS, as during 2007 to 2017 98.2% of newborns was screened within this window (96.9% within 72‐168 hours after birth). One percent of the newborns was screened <72 hours. However, the main reason to perform a screening this early is severe illness, resulting in unrepresentative results. Therefore, these samples were not included in the study. Exclusion criteria were: carnitine >100 μmol/L, unreliable quality of DBS based on registered remarks on the DBS card and GA >308 days. Missing data were handled by listwise deletion, as the data were missing at random (Supplementary Figure S1) and consisted of <0.07% of cases. Finally, records of all repeat samples from individual neonates were excluded from further analysis to avoid selection bias towards low carnitine concentrations, as repeat samples are often taken when the carnitine concentration in first sample is ≤5 μmol/L.

Newborns were divided into groups based on their GA at birth: preterm: ≤27^+6^ weeks (GA_<28_), 28 ≤ 29^+6^ weeks (GA_28‐30_), 30 ≤ 31^+6^ weeks (GA_30‐32_), 32 ≤ 36^+6^ weeks (GA_32‐37_), term: 37 ≤ 41^+6^ weeks (GA_37‐42_), and finally postterm: 42 ≤ 44 weeks (GA_42‐44_).

Using the standardized Dutch birthweight charts provided by Perined (Dutch perinatal audit and perinatal registration),^10^ included children were categorized according to WfGA into the following categories; SGA (weight ≤10 percentile for GA), appropriate for GA (AGA; weight 10 < 90 percentile for GA), or large for GA (LGA; weight ≥ 90 percentile for GA). Children with a GA exceeding 294 days (42 weeks), were categorized using the standardized birthweights of newborns with a GA of 294 days.

### 2.3 Carnitine analysis in DBS

Carnitine concentrations were determined using 3.2‐mm punches from NBS DBS cards with quantitative electrospray ionization tandem mass spectrometry (ESI‐MS/MS) methods: from January 01, 2007 until October 01, 2008, the Neogram MSMS kit and from October 01, 2008 until December 31, 2017, the NeoBase Non‐derivatized MSMS kit (both kits Perkin Elmer, Turku, Finland—extraction and sample preparation performed according to kit‐insert) in combination with a Waters Micro tandem MS instrument (Waters, Milford, Massachusetts).

Control material at two carnitine concentrations was provided with the kits and was used to monitor daily performance of the assay. The carnitine concentration of the kit controls (approximately 38 and 150 μmol/L) is higher than carnitine in our healthy newborn population (approximately 18.0 μmol/L) and the cutoff values (≤5.0 μmol/L). Acceptable accuracy and precision of the carnitine measurement at the carnitine levels of interest was determined and is regularly assessed using commercially available proficiency testing and quality control material from the Centres for Disease Control and Prevention.

### 2.4 Statistics

Statistical analysis was performed using SPSS (version 25.0.0.2, SPSS IBM, New York, New York) and GraphPad Prism (version 8.00, GraphPad Software, La Jolla, California). Mann‐Whitney *U* tests were used to compare the difference in carnitine concentrations between two groups. The chi‐square test was used for comparison of the amount of children that had a carnitine concentration below 5 μmol/L between groups. Multiple comparisons were adjusted for using the Bonferroni correction. Significance was assumed for *P* < .05.

## 3 RESULTS

**FIGURE 1:**
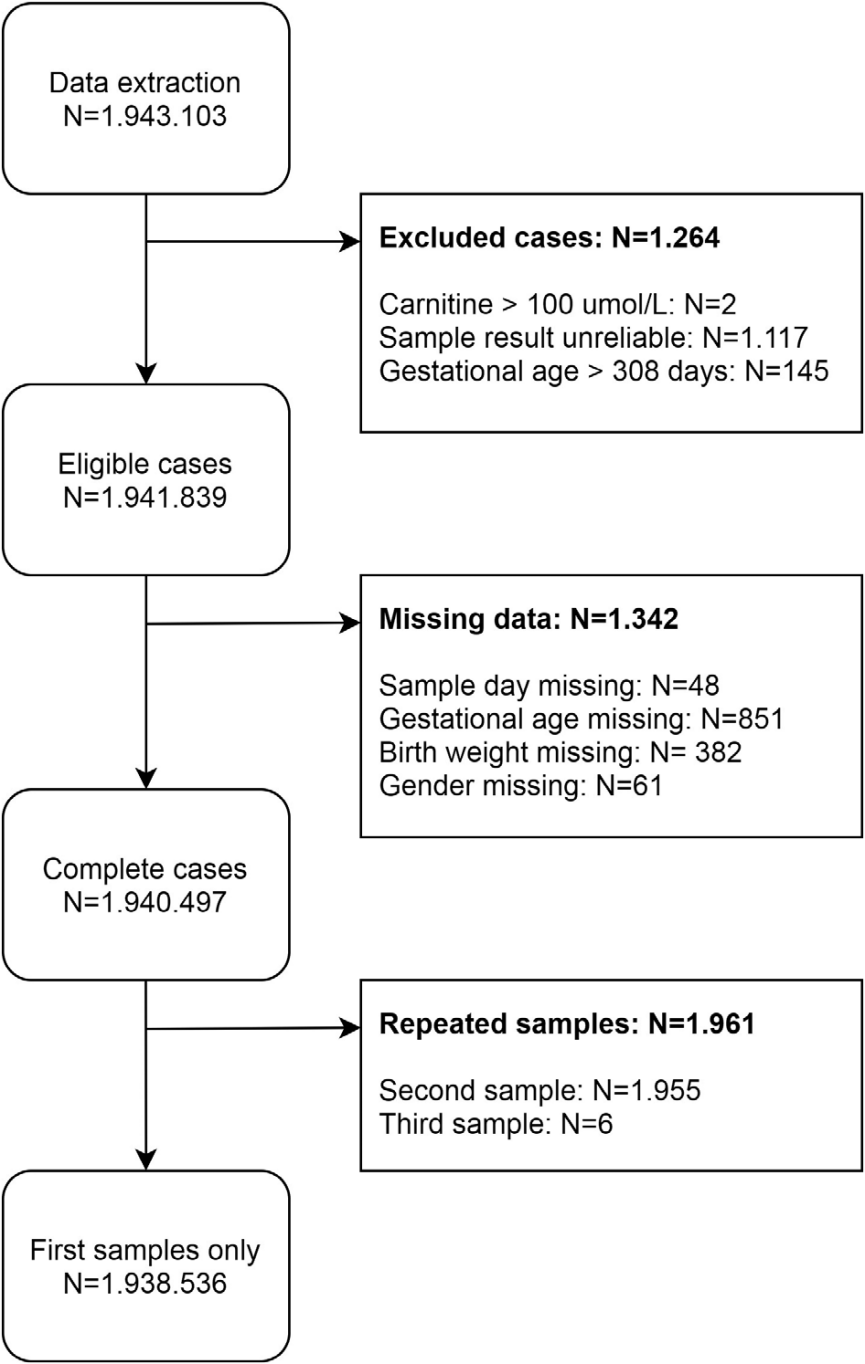
Flowchart of data inclusion

In total, 1.943.103 DBS were available for this study, of which 1.938.536 unique cases (99.8%) were included (Figure 1). Characteristics of the included population are summarized in Table 1. Median age at sampling was 4 days (interquartile range [IQR] 4‐5). The median carnitine concentration of the complete cohort was 18.4 μmol/L (IQR 14.1‐24.0).

**TABLE 1:** Baseline characteristics of newborns included

		Median (IQR)	Range
Gestational age (days)		279 (271‐285)	140‐308
Birthweight (grams)		3450 (3100‐3790)	390‐8000
Sample day (days)		4 (4‐5)	3‐10
Carnitine concentration (μmol/L)		18.4 (14.1‐24.0)	0.0‐99.9
		*N* (%)	
Total		1.938.536 (100)	
Sex	Male	992.770 (51.2)	
	Female	945.766 (48.8)	
Gestational age category	≤27 + 6 wk	4.482 (0.2)	
	28 ≤ 29 + 6 wk	4.849 (0.3)	
	30 ≤ 31 + 6 wk	8.513 (0.4)	
	32 ≤ 36 + 6 wk	113.723 (5.9)	
	37 ≤ 41 + 6 wk	1.766.215 (91.1)	
	42 ≤ 44 wk	40.754 (1.2)	
Weight for gestational age	SGA	226.167 (7.11)	
	AGA	1.508.118 (77.8)	
	LGA	204.251 (10.5)	

Abbreviations: AGA, appropriate for gestational age; IQR, interquartile range; LGA, large for gestational age; SGA, small for gestational age.

### 3.1 Carnitine concentration per group

**FIGURE 2:**
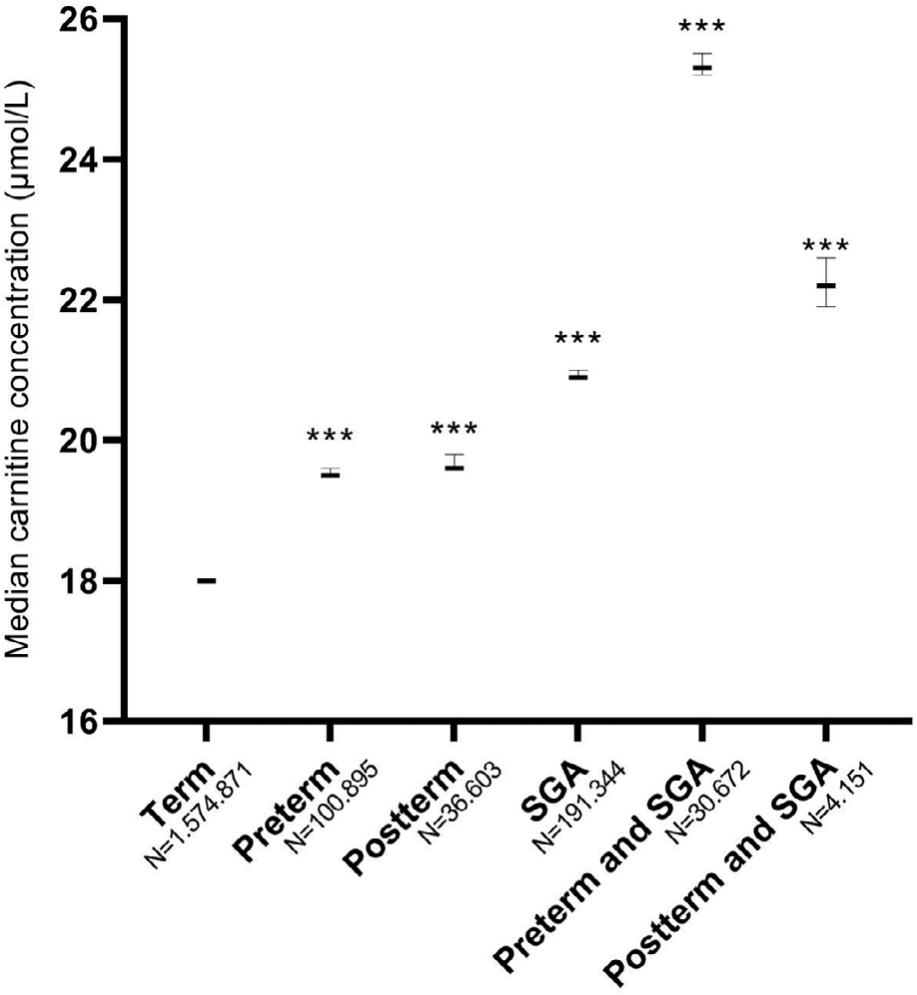
Median carnitine concentrations of preterm, postterm, and/or SGA newborns. All groups were separately compared to term newborns. *P* value was calculated using the Mann‐Whitney *U* test, after Bonferroni correction. SGA, Small for gestational age. Asterisks indicate a *P* value <.001

The median carnitine concentration was significantly higher in all preterm groups, as well as in postterm newborns when compared to term newborns. Median carnitine concentrations were higher in male subjects than female subjects, this was true for all analyzed subgroups (Table S2). Median free carnitine concentrations for all groups are presented in Table 2. Median concentrations were significantly higher in the GA_<28_, GA_28‐30_, and GA_30‐32_ groups compared to term newborns (21.5 [21, 1‐22.0]; 22.9 [3, 6‐23); 24.0 [3, 8‐24] vs 18.2 [4‐18], respectively [μmol/L, 95% confidence interval in parenthesis]). Median free carnitine concentration of all preterm neonates combined was 20.6 (6‐20). Median carnitine concentration in SGA newborns was significantly higher than that in AGA newborns (21.4 [4‐21] vs 18.1 [1‐18]), whereas the median carnitine concentration was significantly lower in LGA newborns (17.3 [3‐17]). To analyze if there was an effect of birthweight on top of the effect of GA, the preterm and postterm groups were separated into a group that was only preterm/postterm and a group that was both preterm/postterm and SGA (Figure 2). Carnitine concentrations were 25.3 (2‐25) in newborns that were both preterm and SGA vs 19.5 (5‐19) in normal weight preterm babies and 22.2 (6, 9‐22) in newborns that were both postterm and SGA vs 19.6 (6‐19) in normal weight postterm babies, indicating an additive effect of birthweight and GA. The 1st percentile and 99th percentile were calculated for all groups and are described in Table 2.

**TABLE 2:** Overall median carnitine concentrations

		Carnitine concentration (μmol/L)			Percentile	
		Median	95% CI	*P* value^a^	1st	99th
Total		18.4	18.4‐18.5	‐	7.3	48.60
Sex	Male	19.0	19.0‐19.1	<.001	7.10	45.60
	Female	17.8	17.8‐17.9		7.60	51.00
Gestational age category	GA_<28_	21.5	21.1‐22.0	<.001	6.50	64.00
	GA_28‐30_	22.9	22.6‐23.3	<.001	7.70	65.50
	GA_30‐32_	24.0	23.8‐24.3	<.001	8.50	66.50
	GA_32‐37_	20.2	20.2‐20.3	<.001	8.00	58.00
	GA_37‐42_	18.2	18.2‐18.3	‐	7.30	47.30
	GA_42‐44_	19.8	19.7‐20.0	<.001	7.50	51.10
Weight for gestational age	SGA	21.4	21.4‐21.5	<.001	8.40	59.40
	AGA	18.1	18.1‐18.2	‐	7.30	46.30
	LGA	17.3	17.3‐17.4	<.001	7.00	45.80
Gestational age and SGA compiled	Term, non‐SGA	18.0	18.0‐18.1	‐	7.20	45.60
	Preterm, non‐SGA	19.5	19.5‐19.6	<.001	7.70	52.90
	Postterm, non‐SGA	19.6	19.6‐19.8	<.001	7.40	50.00
	Term and SGA	20.9	20.9‐21.0	<.001	8.30	56.10
	Preterm and SGA	25.3	25.2‐25.5	<.001	9.20	69.80
	Postterm and SGA	22.2	21.9‐22.6	<.001	8.40	58.20

*Note*: Gestational age categories compared against term category. SGA and LGA compared against AGA. Compiled subgroups were compared to term, non‐SGA newborns.

Abbreviations: AGA, appropriate for gestational age; CI, confidence interval; LGA, large for gestational age; SGA, small for gestational age.

a Mann–Whitney *U* test after Bonferroni correction.

### 3.2 Day‐to‐day carnitine concentrations per group

**FIGURE 3:**
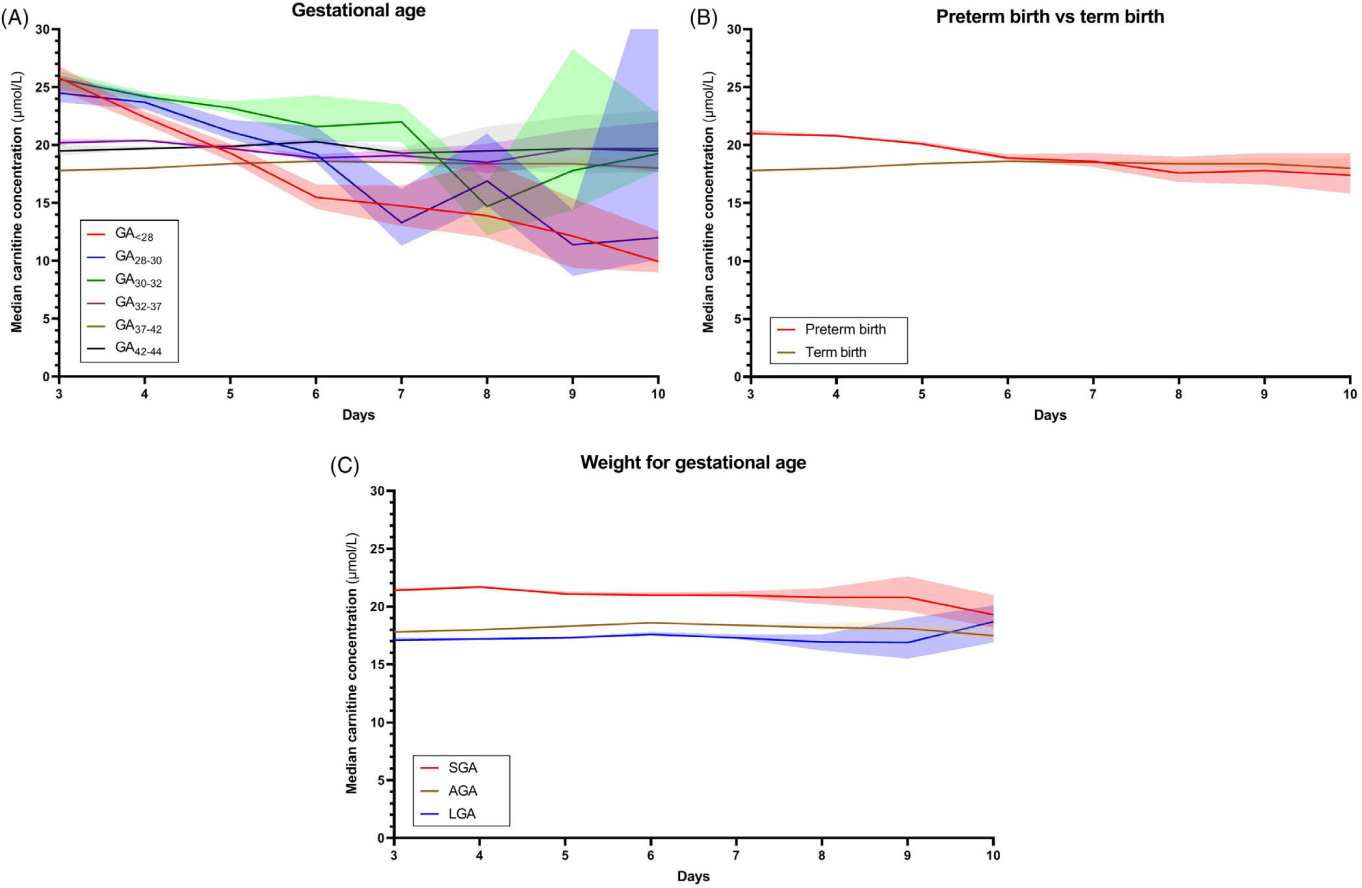
Median carnitine concentration course. Median carnitine concentration courses from days 3 to 10 in groups of various, A, gestational age, B, gestational age with preterm births compiled, compared to term births and, C, weight for gestational age. Transparent areas represent 95% confidence intervals. AGA, appropriate for gestational age; LGA, large for gestational age; SGA, small for gestational age

Carnitine concentrations in the first 10 days of life in all groups are presented in Figure 3. On the third day of life, GA_<28_, GA_28‐30_, and GA_30‐32_ showed the highest median carnitine concentrations (25.8, 24.5, and 25.7 μmol/L, respectively) vs 17.8 μmol/L in term infants. In the preterm children, median carnitine concentration in groups GA_<28_ and GA_28‐30_, intersect median carnitine of term newborns at days 5 and 6, respectively, ultimately reaching a median carnitine concentration of 9.9 and 12.0 μmol/L at day 10, respectively. Medians of GA_32‐37_ and GA_42‐44_ were mildly elevated compared to term newborns throughout the entire period. To assess the effect of prematurity in a broader sense, all preterm groups were compiled (GA ≤36^+6^ weeks) in Figure 3B, revealing an initially increased median carnitine concentration (21.0 μmol/L), approximating the median of term newborns at day 6.

**FIGURE 4:**
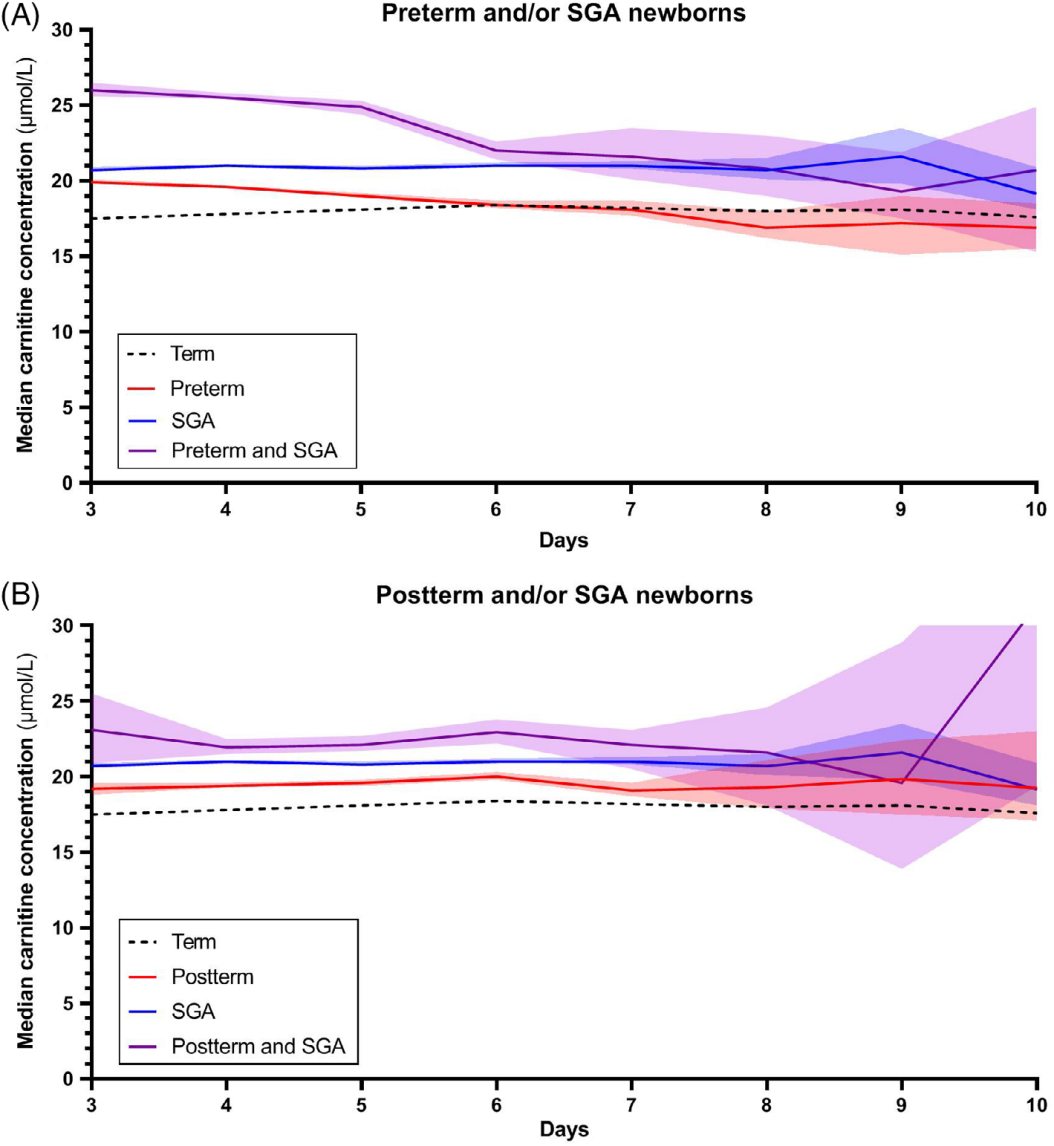
Median carnitine concentration course. Median carnitine concentration courses from days 3 to 10 in, A, preterm and/or SGA newborns and, B, postterm and/or SGA newborns. Transparent areas represent 95% confidence intervals. SGA, small for gestational age

These changes in median carnitine concentrations during days 3 to 10 were not observed within groups of differing WfGA (Figure 3C), where carnitine concentration remained more stable over time. Median carnitine in SGA newborns was roughly 2.8 μmol/L higher than in AGA newborns, whereas the median in LGA newborns was approximately 0.7 μmol/L lower than in AGA newborns. The median carnitine course of the combined groups is presented in Figure 4. It shows, again, a cumulative effect when both attributes are present.

### 3.3 Analysis of carnitine concentration threshold

To analyze whether the relatively high carnitine concentrations in newborns with differing GA or WfGA influenced the detection rate of carnitine deficiency, we determined the number of children who had a NBS carnitine level below the screening cutoff value of 5 μmol/L (Table 3). The percentage of newborns with a carnitine ≤5 μmol/L was 0.062% for the entire cohort. Newborns with such a low carnitine level were significantly underrepresented in the group with a GA between 32 and 37 weeks as well as even more pronounced in the group of newborns that were SGA. Interestingly, extremely preterm infants were significantly overrepresented (0.201%). However, for the newborns in this group that had carnitine concentration below the threshold, the median sample day was day 7 (IQR 6‐9) (data not shown), when carnitine concentrations have already decreased (Figure 3A). In postterm children and children who are LGA, carnitine concentrations below the threshold were found more often than in the entire population (0.083% and 0.096%, respectively).

**TABLE 3:** Number of newborns with carnitine concentration below cutoff value

	Count (N)				Sample day (N)
	Carnitine ≤5 μmol/L	Carnitine >5 μmol/L	% ≤ 5 μmol/L	*P* value^a^	Median (IQR)
Total	1.179	1.937.357	0.061	‐	4 (4‐5)
GA_<28_	9	4.473	0.201	.**0010**	4 (4‐5)
GA_28‐30_	4	4.845	0.083	2.811	4 (4‐5)
GA_30‐32_	3	8.510	0.035	1.626	4 (4‐4)
GA_32‐37_	38	113.685	0.033	**.0008**	4 (4‐4)
GA_37‐42_	1.091	1.765.124	0.062	‐	4 (4‐5)
GA_42‐44_	34	40.720	0.083	.4156	4 (4‐5)
SGA	56	226.111	0.025	**<.0001**	4 (4‐5)
AGA	927	1.507.191	0.062	‐	4 (4‐5)
LGA	196	204.055	0.096	**<.0001**	4 (4‐5)
Term. non‐SGA	1.038	1.573.833	0.066	‐	4 (4‐5)
Preterm, non‐SGA	51	100.844	0.051	.3173	4 (4‐4)
Postterm, non‐SGA	34	36.569	0.093	.2391	4 (4‐5)
Term and SGA	53	191.291	0.028	**<.0001**	4 (4‐5)
Preterm and SGA	3	30.669	0.010	**.0007**	4 (4‐4)
Postterm and SGA	0	4.151	0.000	.4900	4 (4‐5)

*Note*: The amount and corresponding percentage of children that had a carnitine ≤5 μmol/L in primary blood sample in groups based on gestational age and/or weight for gestational age. Gestational age categories compared against term category. SGA and LGA compared against AGA. Compiled subgroups were compared to term, non‐SGA newborns.

Abbreviations: AGA, appropriate for gestational age; Carnitine, carnitine concentration; IQR, interquartile range; LGA, large for gestational age; SGA, small for gestational age.

a Chi‐square test after Bonferroni correction.

## 4 DISCUSSION

To our knowledge, this is the first study to evaluate the pattern in carnitine concentrations during the first 10 days of life in relation to GA and WfGA. We demonstrate that carnitine concentration in preterm, postterm, and SGA newborns is higher compared to term newborns on the third day of life. In addition, our data show that on the sixth day of life, median carnitine concentrations of premature infants approximate those of term infants, whereas the concentrations of postterm and SGA infants remain elevated compared to AGA term infants. This corresponds with previously reported data.^2^, ^3^, ^4^, ^5^, ^6^, ^7^, ^8^, ^9^

We evaluated whether the relatively increased median carnitine concentrations in certain groups lead to a lower number of referrals for a free carnitine ≤5 μmol/L. Indeed, we found that relatively fewer SGA infants and children born after 32 to 37 weeks of gestation were referred.

The reason for the initially relatively higher concentrations of carnitine in these groups is not fully understood. It is plausible that placental transport of carnitine is facilitated in a phase where fetuses are not yet capable of supporting their own carnitine demand, resulting in relatively high carnitine concentrations in preterm infants that are born during this phase.^11^ Previous research demonstrated a crucial role for fatty acid oxidation in fetal development and in late pregnancy, highlighting the need for carnitine in these stages.^12^, ^13^, ^14^, ^15^, ^16^ In addition, renal development is incomplete in extremely preterm infants, resulting in an increased postpartum renal wasting of carnitine.^17^, ^18^ This is reflected by the finding of a relatively rapid decrease of median carnitine concentrations in extremely premature newborns compared to late preterm newborns (Figure 3A). Theoretically, the elevated carnitine levels in preterms might also be caused by overestimation of the measured carnitine level in DBS. It is known that a higher hematocrit can increase certain metabolite concentrations when measured in DBS, thus giving rise to higher carnitine levels in infants that are prone to higher hematocrit levels, such as SGA and postterm infants.^19^, ^20^, ^21^, ^22^ However, preterm children are known to have lower hematocrit at birth; the relatively higher carnitine concentration in this group, make this a less likely explanation.^21^, ^22^

The sampling window employed in the Dutch NBS program is rather wide, which made it possible to analyze the concentration course through several days of life. These findings could aid countries where the screening interval exceeds 71 hours.^23^ For programs that perform earlier screening, our results may also be relevant when screening is conducted on a second DBS.

Several countries use free carnitine as a marker for PCD in NBS. Though the Dutch NBS program does not target PCD, some cases were identified after referral due to repeated low carnitine in DBS. At the time of writing, feasibility of implementation of PCD in the Dutch NBS is being investigated through a nationwide study. The results from the current study can be used to better define an appropriate cutoff for detection of this disease, for example, through variable dependent cutoff ranges. However, clinical data and information on false‐positive and false‐negative rates are required to design a specific referral strategy. Until such a strategy is defined, our results help physicians get a grasp on the physiological carnitine concentration course, which they can utilize when investigating referred newborns.

It is possible to automatically adjust for covariates with the use of Collaborative Laboratory Integrated Reports (CLIR). CLIR is a multivariate pattern recognition software and interactive web tool, which uses a database containing 60 different NBS conditions, .8 million reference profiles and 16.363 true and false‐positive cases collected from 239 NBS programs in 70 countries (as of January 03, 2020). With these data, CLIR is able to accurately create moving percentiles, adjusted for the variables GA, birthweight, sample day, and sex. CLIR eliminates the need for multiple variable‐dependent reference ranges and replaces such ranges with a single adjusted score, reflecting the likelihood of a given disease being present. However, for programs currently unable to use CLIR in the routine interpretation of NBS results, the findings of this study indicate that variable‐dependent reference ranges are desirable in order to accurately evaluate carnitine concentrations in NBS.

By using data from the Dutch NBS program, large sample sizes could be reached for all groups, making a robust analysis of the effects of all attributes on the median carnitine concentration feasible. However, this study has some limitations. Since data were collected anonymously, it was not possible to confidently determine if all cases were healthy individuals. As illness is known to lower carnitine concentrations, we attempted to minimize the impact of this confounder by excluding samples that probably originated from ill individuals.^24^ Still, it is probable that at least part of our data concerns ill newborns, with a majority likely in the preterm group. We believe this does not alter our conclusion, since, despite the presence of this potential confounder, we still found higher carnitine concentrations in preterm groups. Another limitation to our study is the lack of information on the newborns nutrition. As many formulae contain carnitine, they could serve as an exogenous source of carnitine in those that have received it. Contrarily, parenteral nutrition does not contain carnitine and would therefore deplete carnitine stores if not supplemented.^25^ It should be noted that in the Netherlands, it is not common practice to supplement carnitine in preterm infants in conjunction with parenteral feeding during the first month of life. However, as information on nutrition was not available in our dataset, we could not fully exclude that infants were supplemented carnitine.

In conclusion, median carnitine concentrations in DBS of newborns vary with day of sampling, GA, and WfGA. It is important to take these covariates into account when interpreting NBS results.

## CONFLICT OF INTEREST

The authors declare no potential conflicts of interest.

## AUTHOR CONTRIBUTIONS

Loek L. Crefcoeur, Gepke Visser, and Rendelien K. Verschoof‐Puite designed the research. Loek L. Crefcoeur analyzed the data. Monique G. M. de Sain‐van der Velden, Sacha Ferdinandusse, Mirjam Langeveld, Rose Maase, Frédéric M. Vaz, Ronald J. A. Wanders, and Frits A. Wijburg gave valuable input for interpretation of the data and reviewed the manuscript. Loek L. Crefcoeur, Gepke Visser, Rendelien K. Verschoof‐Puite, and Peter J. C. I. Schielen wrote the paper.

## ETHICS STATEMENT

Ethics approval was not required, as no identifying information was acquired. Data retrieval was approved by the Commission Data applications Praeventis of the RIVM‐DVP.

## Supporting information


**FIGURE S1** Marginplots missing data

**FIGURE S2** Median carnitine concentrations

**TABLE S1** Overview of previous research on postnatal carnitine concentrations

**TABLE S2** Subanalysis of median carnitine concentrations on sex
