# Supplementary figures and images for: Neonatal carnitine concentrations in relation to gestational age and weight
Source: JIMD Rep. 2020 Sep 8;56(1):95–104. doi: 10.1002/jmd2.12162 (PMC7653253; doi:10.1002/jmd2.12162)

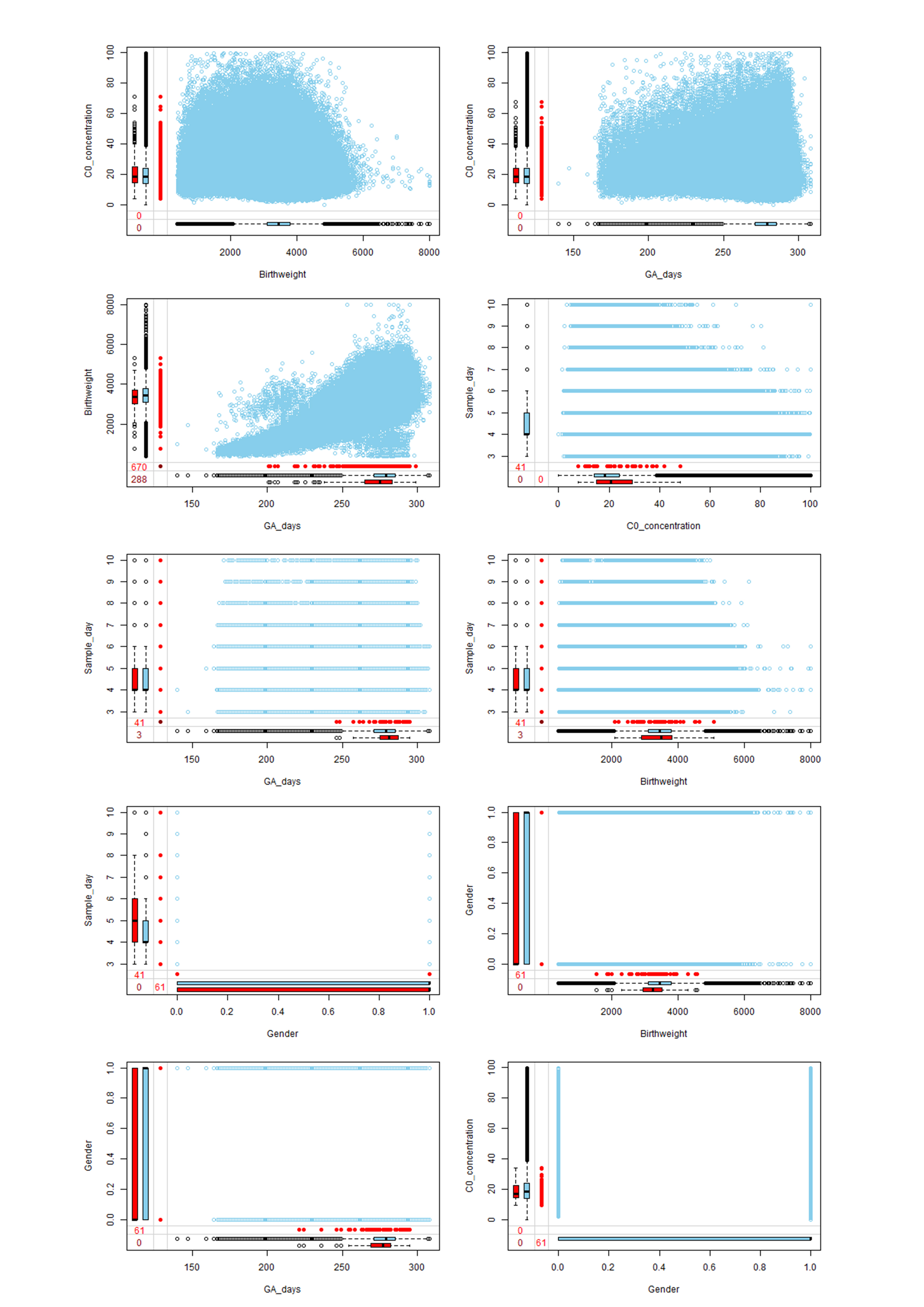

Supplement: Supplementary file 1 — FIGURE S1 Marginplots missing data [file JMD2-56-95-s001.jpg]

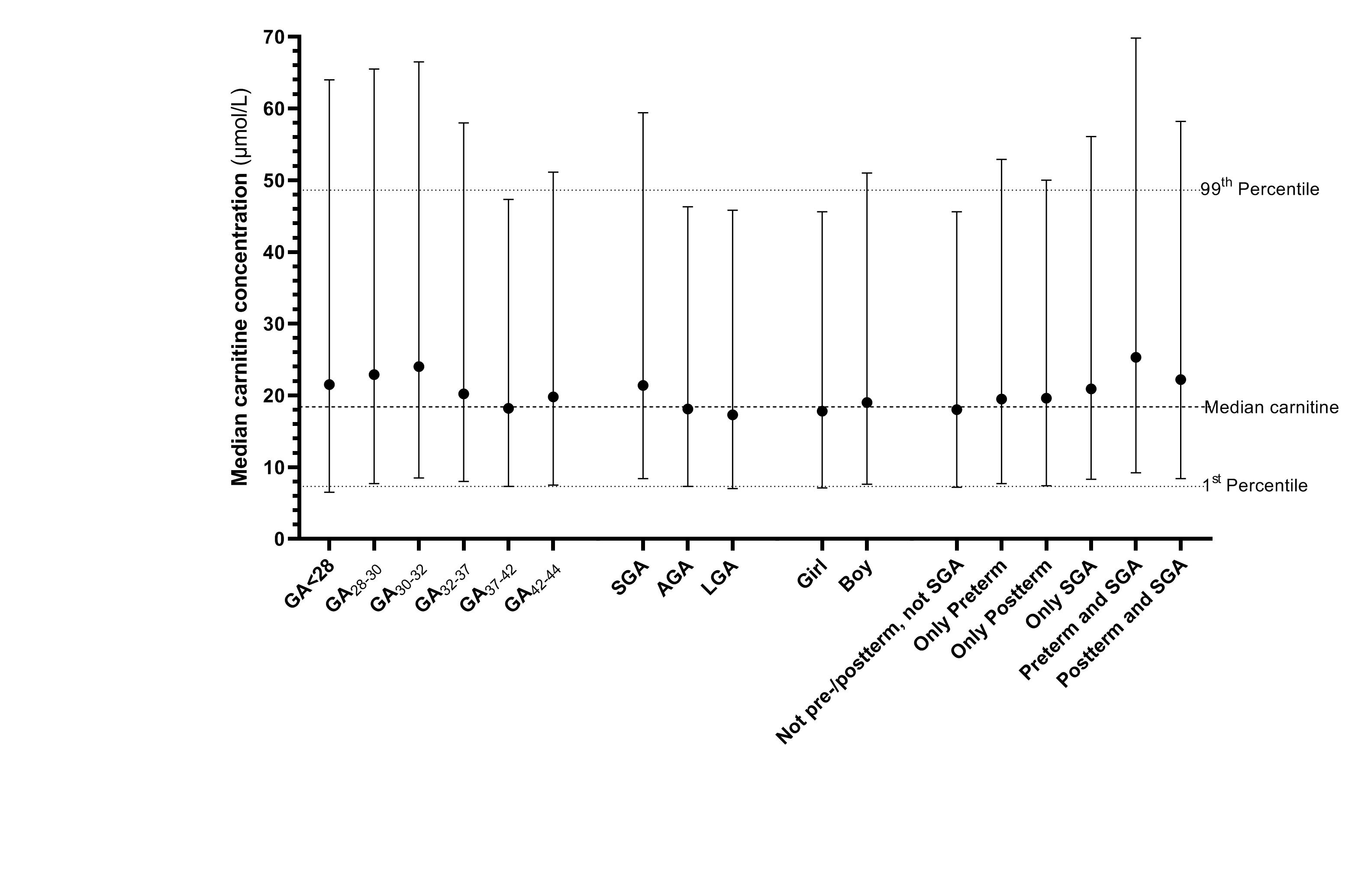

Supplement: Supplementary file 2 — FIGURE S2 Median carnitine concentrations [file JMD2-56-95-s002.jpg]
