# Supplementary material for: Neonatal carnitine concentrations in relation to gestational age and weight
Source: JIMD Rep. 2020 Sep 8;56(1):95–104. doi: 10.1002/jmd2.12162 (PMC7653253; doi:10.1002/jmd2.12162)
Supplement: Supplementary file 3 — TABLE S1 Overview of previous research on postnatal carnitine concentrations [file JMD2-56-95-s003.docx]

**Supplementary Table 1:** Overview of previous research on postnatal carnitine concentrations in (A) preterm vs term newborns (B), SGA vs AGA newborns.

**A**

|  | Term | | Preterm | | |  |  |
| --- | --- | --- | --- | --- | --- | --- | --- |
| Author | N | Carnitine Concentration  (µmol/L)* | Preterm definition  (Weeks of Gestation) | N | Carnitine concentration  (µmol/L)* | Sample day  (days) | Matrix |
| Battistella et al. 1980 | 15 | 31.2 ± 2.5 (SE) | 30-32 | 29 | 43.0 ± 5.6 (SE) | 0 | Cord blood |
|  |  |  | 33-36 |  | 37.5 ± 3.1 (SE) |  |  |
| Shenai et al. 1983 | 72 | 22.4 ± 0.8 (SE) | ≤36 | 53 | 29.0 ± 1.8 (SE) | 0 | Cord blood |
| Watkins et al. 2019 | 150 | 19.9 (IQR 6.3-29.5) | 23-36 | 150 | 21.7 (IQR 12.3-32.1) | 0 | Cord blood |
| Novak et al. 1981 | 7 | ~5.0 ± 2.5 (SE) | 30-37 | 10 | ~16.0 ± 4.0 (SE) | 0 | Cord blood |
| Mandour et al 2013 | 143 | 26.3 (IQR 18.9-33.9) | 27-36 | 131 | 30.0 (IQR 22.4-42.9) | 5 | DBS |
|  |  | - |  |  | 21.5 (IQR 16.1-30.2) | 14 | DBS |
| Liu et al. 2017 | 8914 | 15.5 (p5-95: 9.2-26.4) | <37 | 65 | 18.2 (p5-p95: 10.7-32.4) | 3 | DBS |
| Meyburg et al. 2002 | 30 | 28.0 ± 10.0 (SD) | 22-27 | 30 | 41.0 ± 17.0 (SD) | 5 | DBS |
|  |  |  |  |  | ~35.0 ± 8.0 (SE) | 7 |  |
|  |  |  |  |  | ~20.0 ± 4.0 (SE) | 14 |  |
|  |  |  | 28-31 | 30 | 34.0 ± 12.0 (SD) | 5 | DBS |
|  |  |  |  |  | ~27.0 ± 2.0 (SE) | 7 |  |
|  |  |  |  |  | ~37.0 ± 3.0 (SE) | 14 |  |
|  |  |  | 32-36 | 30 | 37.0 ± 15.0 (SD) | 5 | DBS |
| * Mean ± SE/SD or Median (IQR) or Median (p5-p95)  ~ exact data not provided, data estimated from published graphs. | | | | | | |  |

|  | AGA | | SGA | |  |
| --- | --- | --- | --- | --- | --- |
| Author | N | Carnitine Concentration  (µmol/L)* | N | Carnitine concentration  (µmol/L)* | Sample day  (days) |
| Sánchez-Pintoz et al. 2016 | 73** | 25.7 ± 16.0 | 71** | 32.2 ± 14.4 | 3-5 |
|  |  | 20.8 ± 11.2 |  | 23.5 ± 12.2 | 15 |
|  |  | 24.9 ± 16.2 |  | 25.0 ± 13.6 | 30 |
|  |  | 36.1 ± 19.5 |  | 30.4 ± 9.5 | 40 |
| Liu et al. 2017 | 8264 | 15.4 ( p5-95: 9.2-26.2) | 715 | 16.4 ( p5-95: 9.7-28.5) | 3 |
|  | | | | | |
| * Mean ± SD or Median (p5-p95)  ** not all sample days included the complete cohort. Total cohort size: day 3-5, N=108; day 15, N=81; day 30, N=57; day 40, N=17. | | | | | |

**B**
