# Supplementary material for: Neonatal carnitine concentrations in relation to gestational age and weight
Source: JIMD Rep. 2020 Sep 8;56(1):95–104. doi: 10.1002/jmd2.12162 (PMC7653253; doi:10.1002/jmd2.12162)
Supplement: Supplementary file 4 — TABLE S2 Subanalysis of median carnitine concentrations on sex [file JMD2-56-95-s004.docx]

|  |  |  |  | Sex | |  | Carnitine concentration (µmol/L) | | | |  |
| --- | --- | --- | --- | --- | --- | --- | --- | --- | --- | --- | --- |
|  |  |  |  | Male | |  | Male | | Female | |  |
|  |  |  |  | N | (%) |  | Median | 95% CI | Median | 95% CI |  |
|  | Total |  |  | 992.770 | (51,2) |  | 19,0 | 19,0-19,1 | 17,8 | 17,8-17,9 |  |
|  | Gestational age category | GA_<28_ |  | 2.446 | (54,6) |  | 22,8 | 22,3-23,3 | 20,0 | 19,6-20,6 |  |
|  |  | GA_28-30_ |  | 2.624 | (54,1) |  | 24,4 | 23,8-25,0 | 21,7 | 21,2-22,1 |  |
|  |  | GA_30-32_ |  | 4.631 | (54,4) |  | 24,8 | 24,4-25,1 | 23,2 | 22,9-23,6 |  |
|  |  | GA_32-37_ |  | 61.571 | (54,1) |  | 21,0 | 21,0-21,1 | 19,3 | 19,3-19,4 |  |
|  |  | GA_37-42_ |  | 900.271 | (51,0) |  | 18,9 | 18,9-19,0 | 17,6 | 17,6-17,7 |  |
|  |  | GA_42-44_ |  | 21.227 | (52,1) |  | 20,4 | 20,3-20,6 | 19,1 | 19,0-19,3 |  |
|  | Weight for gestational age | SGA |  | 114.415 | (50,6) |  | 22,4 | 22,4-22,5 | 20,5 | 20,5-20,6 |  |
|  |  | AGA |  | 773.113 | (51,3) |  | 18,8 | 18,8-18,9 | 17,5 | 17,5-17,6 |  |
|  |  | LGA |  | 105.242 | (51,5) |  | 17,9 | 17,9-18,0 | 16,7 | 16,7-16,8 |  |

**Supplementary Table 2. Subanalysis of median carnitine concentrations on sex.** Median carnitine concentrations for gestational age groups and weight for gestational age. Stratified based on sex.
